# The hereditary spastic paraplegia type 21 (SPG21) protein is a RAB7A effector that promotes noncanonical mTORC1-catalyzed TFEB phosphorylation and cytoplasmic retention

**DOI:** 10.1091/mbc.E25-07-0346

**Published:** 2025-09-12

**Authors:** Jennifer M. Kunselman, Chad D. Williamson, Adriana E. Golding, Rui Jia, Mira Sohn, Ryan K. Dale, Juan S. Bonifacino

**Affiliations:** ^a^Division of Neurosciences and Cellular Structure, Eunice Kennedy Shriver National Institute of Child Health and Human Development, National Institutes of Health, Bethesda, MD 20892; ^b^Bioinformatics and Scientific Programming Core, Eunice Kennedy Shriver National Institute of Child Health and Human Development, National Institutes of Health, Bethesda, MD 20892; Children's Hospital of Philadelphia

## Abstract

Hereditary spastic paraplegia type 21 (SPG21) is an inherited neurological disorder caused by biallelic mutations in the *SPG21* gene, which encodes a protein named SPG21 or maspardin. Herein, we report that the SPG21 protein localizes to endolysosomes through interaction with the GTP-bound form of RAB7A. Disease-associated *SPG21* variants reduce expression of SPG21 and disrupt its endolysosomal localization in both nonneuronal cells and neurons. Consistent with this localization, functional dependency analysis links SPG21 to endolysosomal and mTORC1 signaling pathways. Biochemical studies reveal that SPG21 depletion does not affect phosphorylation of canonical mTORC1 substrates such as ULK1, S6K1, 4E-BP1, but reduces phosphorylation of the noncanonical mTORC1 substrate TFEB. This enhances nuclear localization of TFEB and expression of a subset of TFEB-target genes. We conclude that SPG21 acts as a RAB7A effector that promotes noncanonical mTORC1-catalyzed phosphorylation of TFEB, thereby suppressing its nuclear localization and transcriptional activity. These findings link SPG21 dysfunction to altered endolysosomal signaling, offering new insights into SPG21 pathogenesis.

## Abstract (executive summary)

- Hereditary spastic paraplegia type 21 is a neurological disorder caused by mutations in the *SPG21* gene, which encodes the protein SPG21 or maspardin. The subcellular localization and physiological role of SPG21 have remained poorly understood.
- Our study reveals that SPG21 localizes to endolysosomes through interaction with the GTP-bound form of RAB7A, where it promotes mTORC1-dependent phosphorylation of the transcription factor TFEB, resulting in its cytoplasmic retention and reduced expression of TFEB target genes.
- These findings identify SPG21 as a novel RAB7A effector and regulator of mTORC1–TFEB signaling, implicating this signaling pathway in the pathogenesis of SPG21-linked hereditary spastic paraplegia.

## INTRODUCTION

Hereditary spastic paraplegias (abbreviated HSPs or SPGs) are a heterogeneous group of inherited neurological disorders characterized by progressive spasticity and weakness of the legs (Blackstone, 2018; Elsayed *et al.*, 2021). Apart from these common characteristics, various types of HSP exhibit differences in clinical presentation, inheritance patterns, and genetic etiology. Pure HSPs present with leg spasticity and weakness, without added neurological manifestations. These symptoms result from axonopathy of upper motor neurons in corticospinal tracts. Complicated HSPs present additional features such as intellectual disability, seizures, ataxia, dementia and/or peripheral neuropathy, arising from defects in other neurons of the nervous system. The inheritance mode of HSPs can be autosomal recessive, autosomal dominant, X-linked, or maternally inherited mitochondrial. To date, mutations in more than 80 genes have been shown to cause HSPs (Blackstone, 2018; Elsayed *et al.*, 2021). These genes encode proteins involved in diverse cellular processes, including protein and organelle transport, endoplasmic reticulum (ER) morphology, mitochondrial function, lipid metabolism, and myelination. In most cases, however, the molecular mechanisms of pathogenesis are poorly understood and treatment remains symptomatic. Thus, there is a crucial need to elucidate how mutations in these genes contribute to HSP.

**FIGURE 1:**
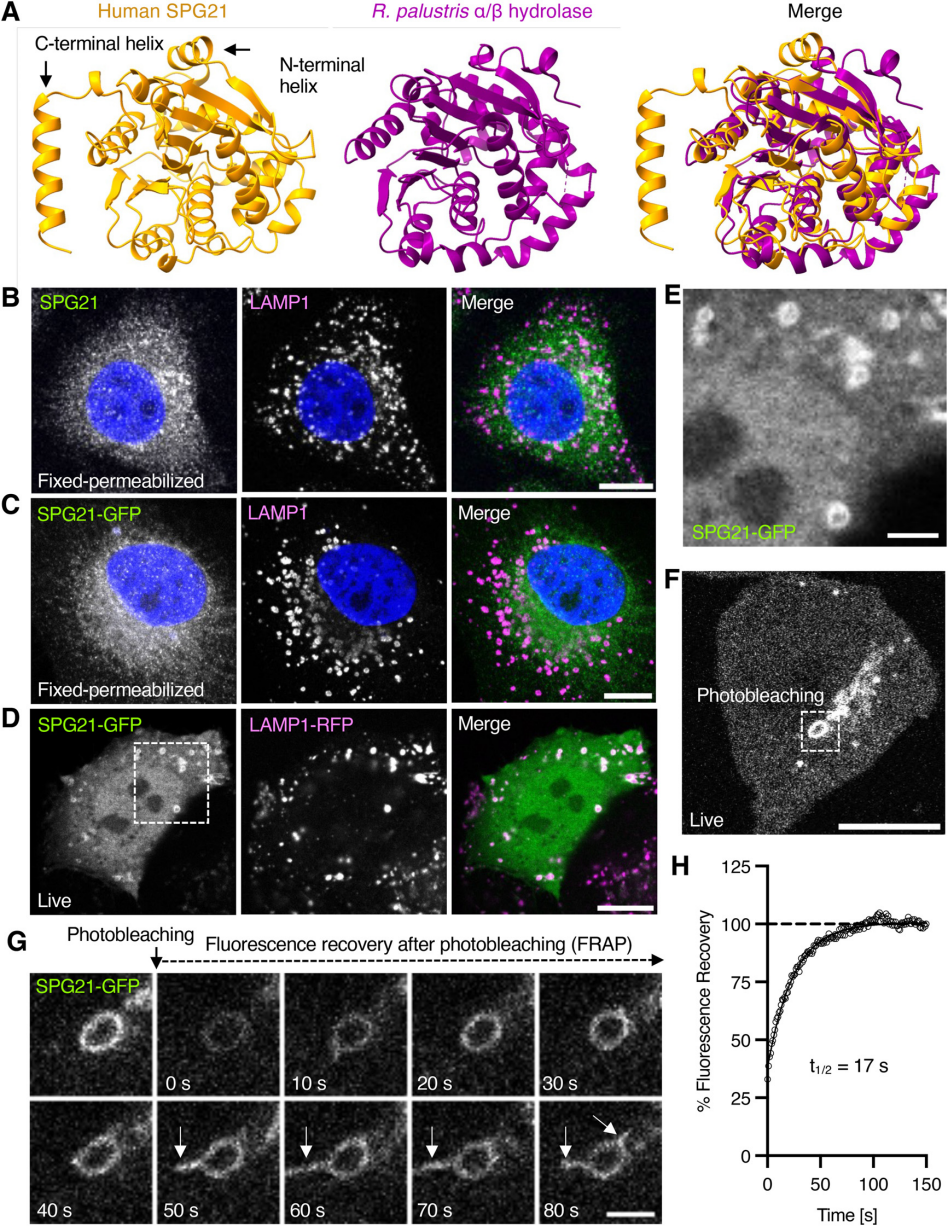
Dynamic localization of SPG21 to endolysosomes. (A) Predicted AlphaFold 3 structure (Abramson *et al.*, 2024) of human SPG21 (orange) and crystal structure of *Rhodopseudomonas palustris* α/β hydrolase (magenta) (PDB entry 4PSU). Additional N-terminal and C-terminal α-helixes in human SPG21 are indicated. (B) HeLa cells were fixed with 4% paraformaldehyde, permeabilized with 0.1% saponin, and immunostained for endogenous SPG21 (green) and LAMP1 (magenta). Nuclei were stained with DAPI (blue). Cells were imaged by confocal fluorescence microscopy; scale bar, 10 µm. (C) HeLa cells were transiently transfected with a plasmid encoding SPG21-GFP, fixed and permeabilized as in B, and immunostained for GFP (green) and endogenous LAMP1 (magenta). Nuclei were stained with DAPI (blue). Cells were imaged by confocal fluorescence microscopy; scale bar, 10 µm. (D) HeLa cells were transiently transfected with plasmids encoding SPG21-GFP (green) and LAMP1-RFP (magenta) and imaged live by confocal fluorescence microscopy; scale bar, 10 µm. (E) Enlargement of box in panel D showing endolysosomes positive for SPG21-GFP; scale bar, 3 µm. (F) HeLa cells were transiently transfected with a plasmid encoding SPG21-GFP and imaged live by confocal microscopy. Boxed area shows an endolysosome that was photobleached for analysis by FRAP; scale bar, 10 µm. (G) Montage of SPG21-GFP FRAP frames acquired at 10-s intervals; scale bar, 2 µm. Arrows indicate tubules emanating from endolysosome. (H) Quantification of SPG21-GFP FRAP from panel F with one-phase association fit.

HSP type 21 (SPG21, also known as “Mast syndrome”) (OMIM 248900) is a complicated, autosomal-recessive form of HSP that was originally described in the North American Old Order Amish (Cross and McKusick, 1967; Simpson *et al.*, 2003), but also occurs in individuals of Japanese, Italian, Chinese, German, and Austrian descent (Ishiura *et al.*, 2016; Schüle *et al.*, 2016; Scarlato *et al.*, 2017; Xue *et al.*, 2021; Amprosi *et al.*, 2022). The first manifestations of this disease can appear in childhood, adolescence, or adulthood in the form of slowly progressing spastic paraplegia. Over time, patients develop cognitive decline, dementia, and extrapyramidal as well as cerebellar signs. Brain magnetic resonance imaging shows that patients have a thin corpus callosum, global brain atrophy and white-matter loss. SPG21 is caused by mutations in the *SPG21* gene (formerly known as *ACP33*) encoding a cytosolic protein of 308 amino acids named SPG21 (also known as ACP33 or maspardin) (Zeitlmann *et al.*, 2001; Simpson *et al.*, 2003). This protein was first identified in a yeast two-hybrid screen for interactors of the cytosolic tail of the T-cell surface glycoprotein CD4 (Zeitlmann *et al.*, 2001). This interaction was proposed to modulate CD4 function independently of the signaling protein tyrosine kinase p56lck (Zeitlmann *et al.*, 2001). However, SPG21 is ubiquitously expressed (https://www.proteinatlas.org/), and its mutations primarily affect the CNS, suggesting that it may have other functions. Homology searches and structural predictions revealed that SPG21 belongs to the superfamily of α/β hydrolase fold proteins (Figure 1A), which are widely distributed across phyla, from bacteria to humans (Ollis *et al.*, 1992). However, SPG21 lacks a catalytic triad typical of most α/β hydrolases (Supplemental Figure S1), suggesting that it does not have enzymatic activity (Simpson *et al.*, 2003). Additionally, SPG21 has an N-terminal and a C-terminal α-helix (Figure 1A).

Homozygous disruption of the *Spg21* gene in mice was shown to cause progressive hind limb dysfunction, consistent with the initial presentation of SPG21 deficiency in humans (Soderblom *et al.*, 2010; Davenport *et al.*, 2016). In addition, *Spg21*-knockout (KO) mice displayed alterations in axon branching and proliferation of cerebral cortical neurons in primary culture, which potentially contribute to the observed neurological phenotypes (Soderblom *et al.*, 2010; Davenport *et al.*, 2016). Immunofluorescence microscopy analyses of cultured cells using an antibody to SPG21 showed distribution of the endogenous protein to the cytosol, *trans*-Golgi network (TGN) and endosomes (Zeitlmann *et al.*, 2001; Hanna and Blackstone, 2009). However, these analyses lacked the sensitivity and resolution to determine a precise localization. Furthermore, they did not demonstrate how SPG21 localizes to those compartments, the impact of SPG21 patient mutations on SPG21 localization, and, ultimately, the function of the protein.

Here, we present findings from an investigation into the localization, membrane-recruitment mechanism, and cellular function of SPG21. Our study reveals that the fixation and permeabilization protocol previously used for immunofluorescence microscopy attenuates the signal for endogenous SPG21, and that improved detection can be achieved by live-cell imaging of SPG21 tagged at its C-terminus with green fluorescent protein (SPG21-GFP). This latter approach allowed us to demonstrate that SPG21-GFP dynamically localizes to late endosomes/lysosomes (henceforth collectively referred to as “endolysosomes”), while showing no visible localization to early endosomes and the TGN. Furthermore, live-cell imaging showed that the association of SPG21 with endolysosomes is dependent on the GTP-bound, active form of the endolysosomal small GTPase RAB7A, but not on other endolysosomal small GTPases such as ARL8 (A and B isoforms), RAB9A, and RRAGB (i.e., RagB). Moreover, coimmunoprecipitation experiments demonstrated that SPG21 preferentially interacts with the GTP-bound form of RAB7A, identifying SPG21 as a *bona fide* RAB7A effector. Additionally, we found that *SPG21* patient variants destabilize the protein and abrogate its association with endolysosomes. Finally, we observed that SPG21 depletion decreases mTORC1-catalyzed phosphorylation of the noncanonical substrate TFEB, enhancing TFEB nuclear localization and transcription of a subset of TFEB target genes. These findings identify SPG21 as a novel RAB7A effector that promotes mTORC1-mediated TFEB phosphorylation and cytoplasmic retention, thereby dampening TFEB-driven transcriptional activity.

## RESULTS

### SPG21 localizes to endolysosomes

To investigate the intracellular localization of SPG21, we initially performed immunofluorescence microscopy of HeLa cells fixed with 4% paraformaldehyde, permeabilized with 0.1% saponin, and immunostained with a commercially available antibody to endogenous SPG21 (Sigma-Aldrich, HPA040407). In agreement with previous studies using a similar protocol (Zeitlmann *et al.*, 2001; Hanna and Blackstone, 2009), we observed a diffuse cytoplasmic distribution of endogenous SPG21, with some perinuclear clusters that roughly co-localized with the endogenous endolysosomal marker LAMP1 (Figure 1B) (Chen *et al.*, 1985; Fermie *et al.*, 2018). Transgenically expressed SPG21-GFP exhibited a similar distribution pattern in fixed-permeabilized HeLa cells (Figure 1C). However, the quality of the images for both endogenous SPG21 and transgenic SPG21-GFP in fixed-permeabilized cells was insufficient to ascertain a specific localization of these proteins. In contrast, live-cell imaging of HeLa cells coexpressing SPG21-GFP and LAMP1-RFP revealed clear colocalization of these proteins to cytoplasmic vesicles characteristic of endolysosomes (Figure 1D). The SPG21-GFP fluorescence appeared ring-like, consistent with SPG21-GFP associating with the limiting membrane of endolysosomes (Figure 1E). These results suggested that the antigenicity or association of SPG21 with endolysosomes is poorly maintained after fixation and permeabilization, and that live-cell imaging is a better method for analysis of SPG21 localization.

To examine the dynamics of SPG21-GFP association with endolysosomes, we performed fluorescence recovery after photobleaching (FRAP) (Lippincott-Schwartz *et al.*, 2018). The assay consisted of photobleaching a region of interest containing endolysosomal SPG21-GFP (Figure 1, F and G) and recording the fluorescence recovery over time (Figure 1, G and H). We observed that the recovery of SPG21-GFP on endolysosomes was rapid (*t*_1/2_ 17 s) and complete (Figure 1, G and H), indicating that SPG21 rapidly cycles between endolysosomal and cytosolic pools. These experiments also revealed that SPG21-GFP associates with both the vacuolar part and tubular projections of endolysosomes (Figure 1G, arrows).

### SPG21 colocalizes with endolysosomal but not early endosomal and TGN small GTPases

**FIGURE 2:**
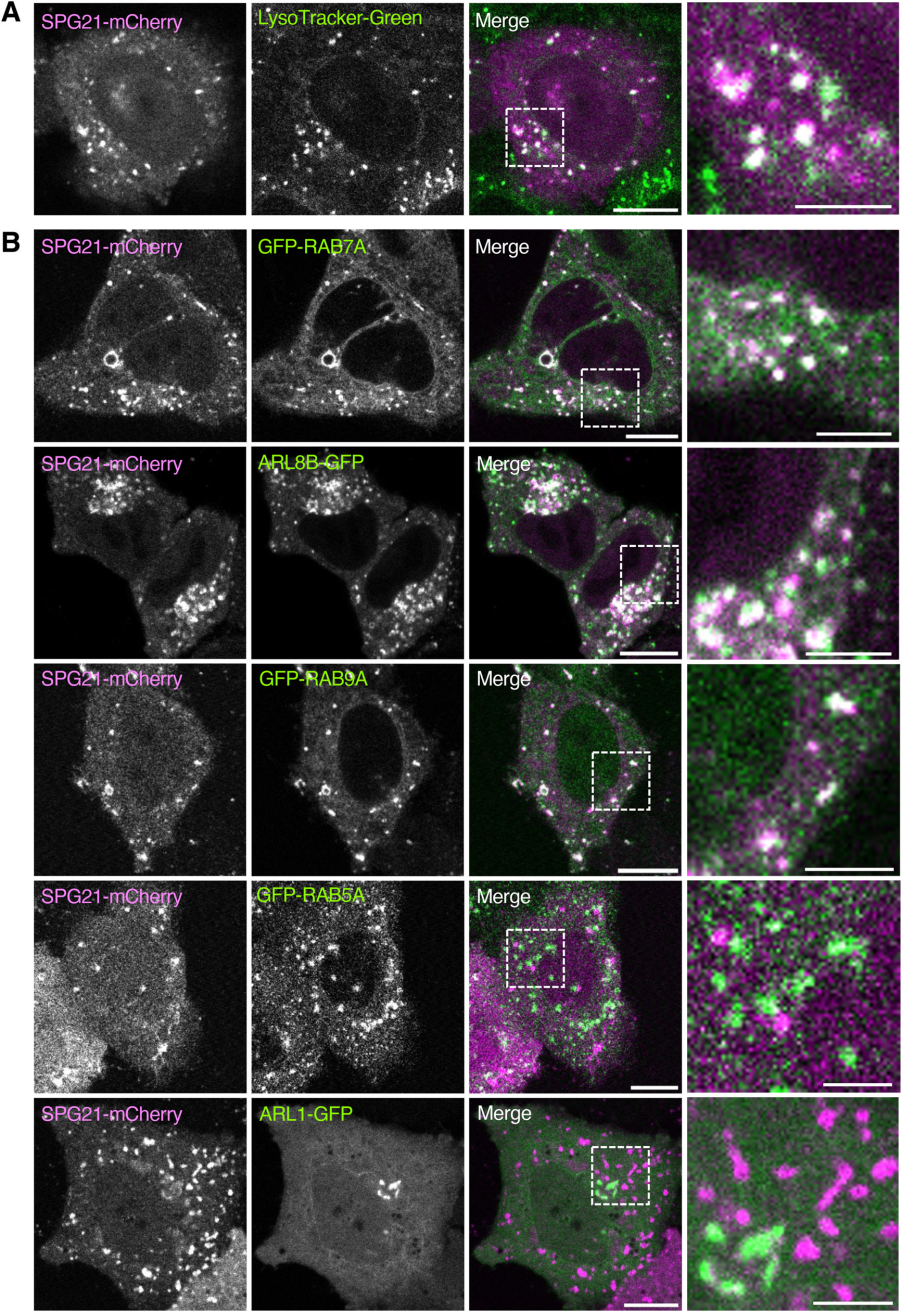
Colocalization of SPG21 with endolysosomal but not early endosomal or TGN markers. (A) HeLa cells were transiently transfected with a plasmid encoding SPG21-mCherry, labeled with LysoTracker-Green (acidic endolysosome marker), and imaged by confocal fluorescence microscopy; scale bars, 10 µm. (B) HeLa cells were transiently cotransfected with plasmids encoding SPG21-mCherry (magenta) and GFP-RAB7A, ARL8B-GFP, GFP-RAB9A (all endolysosomal small GTPases) (green), GFP-RAB5A (early endosomal small GTPase) (green), or ARL1-GFP (TGN small GTPase) (green), and imaged live by confocal fluorescence microscopy; scale bars, 10 µm. Right-most column shows zoomed-in views of boxed areas; scale bars, 4 µm.

To further assess the localization of SPG21, we performed live-cell imaging of HeLa cells expressing SPG21-mCherry and labeled with the acidic endolysosomal marker LysoTracker-Green (Figure 2A), or coexpressed with the small GTPases GFP-RAB7A (Chavrier *et al.*, 1990), ARL8B-GFP (Hofmann and Munro, 2006), GFP-RAB9A (Lombardi *et al.*, 1993) (all endolysosomal markers), GFP-RAB5A (Chavrier *et al.*, 1990) (early endosomal marker), and ARL1-GFP (Lu *et al.*, 2004) (TGN marker) (Figure 2B). We observed a high degree of colocalization of SPG21-mCherry with LysoTracker-Green (Figure 2A), GFP-RAB7A, ARL8B-GFP, and GFP-RAB9A, but not GFP-RAB5A and ARL1-GFP (Figure 2B), confirming the localization of SPG21-mCherry to endolysosomes, and not detectably to early endosomes or the TGN.

### RAB7A is required for recruitment of SPG21 to endolysosomes

Next, we addressed how SPG21 is recruited to endolysosomes. SPG21 lacks any known membrane-binding domains or structural motifs; therefore, its recruitment to endolysosomes most likely involves interaction with a membrane-bound protein. The BioGRID database lists 209 potential interactors for human SPG21 identified in various screens (https://thebiogrid.org/119474/summary/homo-sapiens/spg21.html). Among these potential interactors are RAB7A, ARL8A, ARL8B, RAB9A, and RRAGB, which are known to recruit multiple effectors to endolysosomes (Gillingham *et al.*, 2014; Khatter *et al.*, 2015; Napolitano *et al.*, 2022). Furthermore, human SPG21 was identified as one of several proteins that interact with wild-type (WT) RAB7A in immunopurification–mass-spectrometry and coimmunoprecipitation analyses (McCray *et al.*, 2010). However, none of these GTPase interactions were shown to mediate recruitment of SPG21 to endolysosomes or to be GTP-dependent.

**FIGURE 3:**
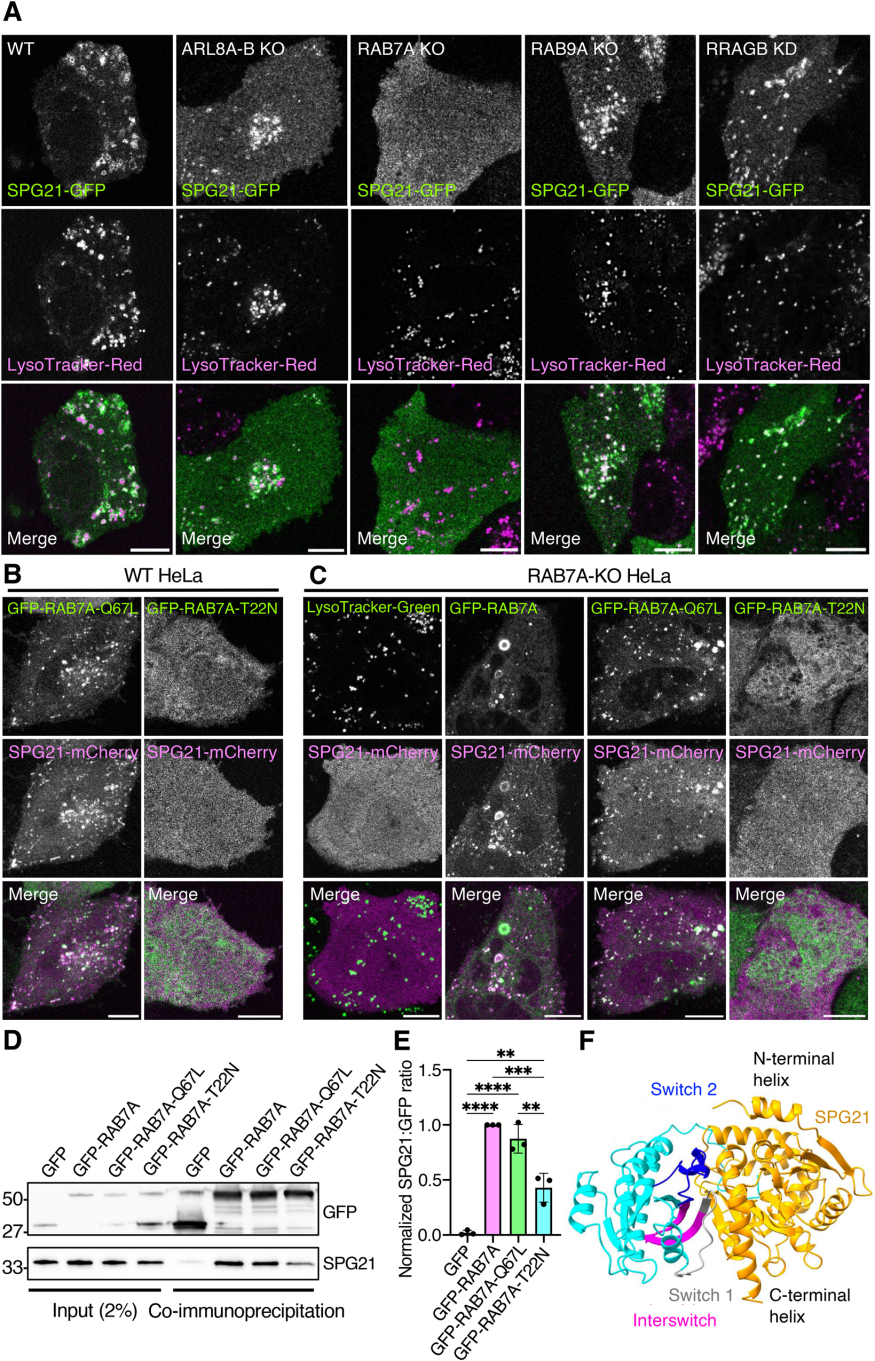
GTP-dependent interaction of RAB7A with SPG21. (A) SPG21-GFP (green) was transiently expressed in WT, ARL8A-B-KO, RAB7A-KO, RAB9A-KO or RRAGB-KD HeLa cells. Cells were labeled with LysoTracker-Red (magenta) and imaged live by confocal fluorescence microscopy; scale bars, 10 µm. (B) WT HeLa cells were transiently cotransfected with plasmids encoding SPG21-mCherry (magenta) and GFP-RAB7A-Q67L or GFP-RAB7A-T22N (green), and imaged live by confocal fluorescence microscopy; scale bars, 10 µm. (C) RAB7A-KO HeLa cells were transiently transfected with plasmids encoding SPG21-mCherry and labeled with LysoTracker-Green, or cotransfected with plasmids encoding SPG21-mCherry and GFP-RAB7A, GFP-RAB7A-Q67L or GFP-RAB7A-T22N (green). Cells were imaged live by confocal fluorescence microscopy; scale bars, 10 µm. (D) HEK293T cells were transiently transfected with plasmids encoding GFP, GFP-RAB7A, GFP-RAB7A-Q67L, or GFP-RAB7A-T22N, and cell extracts were subjected to immunoprecipitation with an antibody to GFP followed by SDS–PAGE and immunoblotting with antibodies to GFP and endogenous SPG21. Positions of molecular mass markers (in kDa) are indicated. (E) Quantification of SPG21 signal relative to GFP from three independent experiments such as that shown in panel D. Values are the mean ± SD. Statistical significance was analyzed by one-way ANOVA with Tukey's multiple comparison test. ****P < 0.0001, ***P < 0.001, **P < 0.01. (F) Structure of human RAB7A bound to human SPG21 predicted by AlphaFold 3 (Abramson *et al.*, 2024).

To determine whether any of these small GTPases are required for endolysosomal recruitment of SPG21, we examined the colocalization of LysoTracker-Red with SPG21-GFP in live HeLa cells with CRISPR-Cas9-induced KO of both the ARL8A and ARL8B paralogues (ARL8A-B), RAB7A, or RAB9A, or siRNA-induced knockdown (KD) of RRAGB (Figure 3A; Supplemental Figure S2). We observed that depletion of ARL8A and ARL8B, RAB9A, or RRAGB had no effect on the association of SPG21-GFP with endolysosomes (Figure 3A). In contrast, RAB7A KO resulted in complete redistribution of SPG21-GFP to the cytosol (Figure 3A). These experiments demonstrated that RAB7A is required for the association of SPG21-GFP with endolysosomes.

### SPG21 preferentially interacts with the GTP-bound form of RAB7A

Like other small GTPases, RAB7A cycles between GTP-bound (active) and GDP-bound (inactive) conformations. Whereas the GTP-bound form of RAB7A is membrane-bound and recruits effectors, the GDP-bound form is cytosolic and is unable to interact with effectors. To determine whether the ability of RAB7A to recruit SPG21 is dependent on the nucleotide state of RAB7A, we coexpressed SPG21-mCherry with constitutively active GFP-RAB7A-Q67L or constitutively inactive GFP-RAB7A-T22N mutants in WT HeLa cells (Figure 3B). Live-cell imaging showed that GFP-RAB7A-Q67L colocalized with SPG21-mCherry on endolysosomes, while GFP-RAB7A-T22N was cytosolic and prevented the association of SPG21-mCherry with endolysosomes (Figure 3B). We also tested whether SPG21-mCherry localization to endolysosomes in RAB7A-KO cells could be rescued by expression of GFP-RAB7A (Figure 3C). We found that WT GFP-RAB7A and GFP-RAB7A-Q67L rescued endolysosomal localization of SPG21-mCherry, whereas GFP-RAB7A-T22N did not (Figure 3C). Taken together, these results demonstrated that SPG21 is recruited to endolysosomes by the GTP-bound form of RAB7A.

Next, we used a coimmunoprecipitation assay to determine if SPG21 physically interacts with RAB7A. HEK293T cells were transfected with GFP (control), WT GFP-RAB7A, GFP-RAB7A-Q67L, or GFP-RAB7A-T22N. Cell extracts were incubated with GFP-Trap beads. Washed beads were analyzed by SDS–PAGE and immunoblotted with antibodies to GFP and endogenous SPG21 (Figure 3D). The SPG21 signal relative to the GFP signal for each condition was quantified from three independent experiments (Figure 3E). We observed that both GFP-RAB7A and GFP-RAB7A-Q67L coimmunoprecipitated SPG21 equally and to a much larger extent than GFP (Figure 3, D and E). In comparison, GFP-RAB7A-T22N coimmunoprecipitated less SPG21 (Figure 3, D and E). Structural analysis using AlphaFold 3 (Abramson *et al.*, 2024) predicted that RAB7A interacts via the switch 1, switch 2, and interswitch regions with the α/β hydrolase fold, but not with the N- and C-terminal α-helixes of SPG21 (Figure 3F), consistent with a small GTPase-effector interaction.

These results demonstrated that SPG21 binds RAB7A in a GTP-dependent manner and is recruited to endolysosomes by RAB7A-GTP, indicating that SPG21 is a RAB7A effector. The fact that GFP-RAB7A and GFP-RAB7A-Q67L behave similarly in all assays additionally suggested that a substantial amount of GFP-RAB7A in the transfected cells exists in the GTP-bound conformation.

### *SPG21* patient mutations decrease expression and abolish endolysosomal localization

**FIGURE 4:**
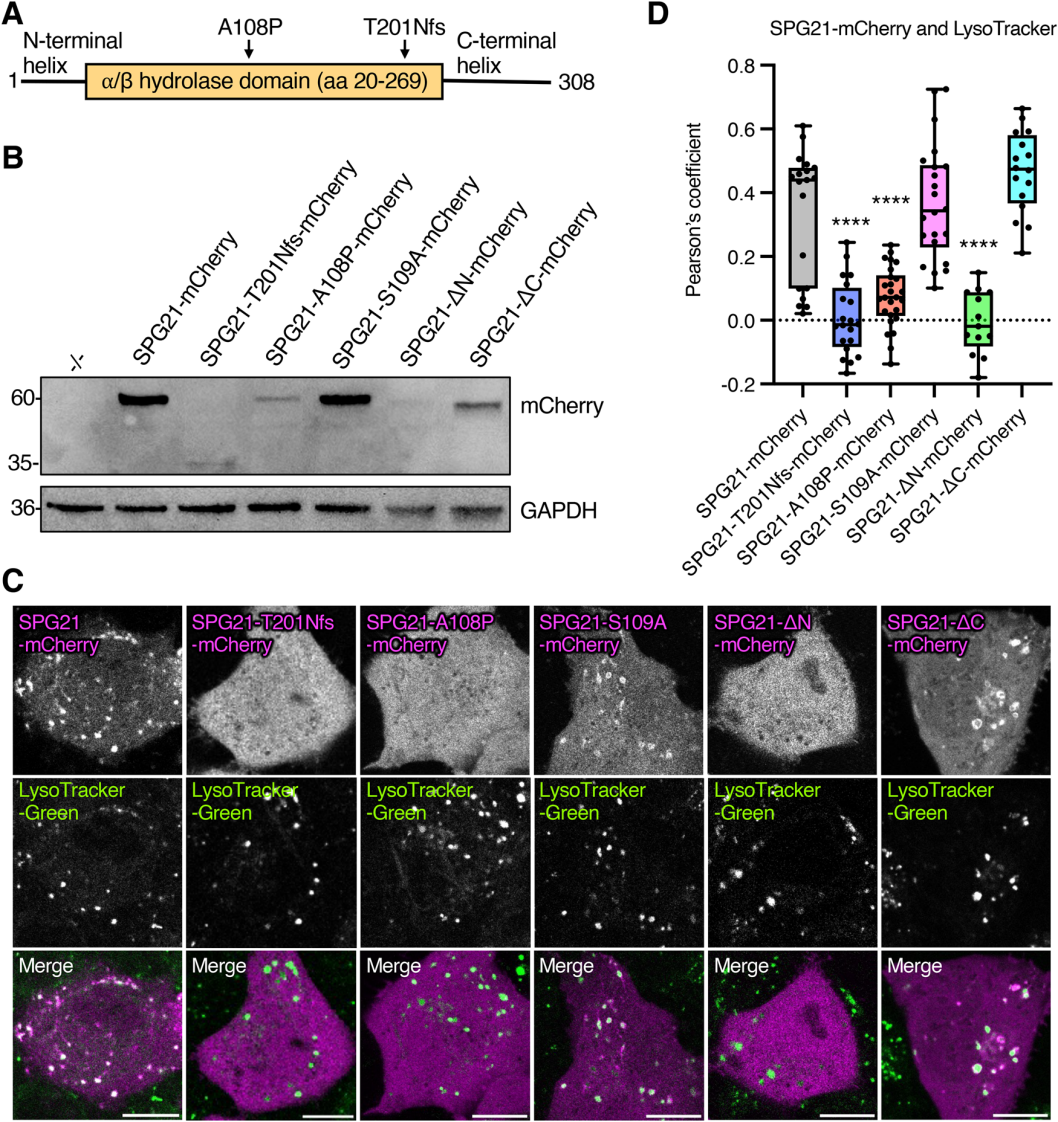
SPG21 patient mutations decrease expression and abolish localization to endolysosomes. (A) Schematic representation of the SPG21 protein, indicating two patient mutations (A108P and pT201Nfs) within the α/β hydrolase domain, and the N-terminal and C-terminal α-helixes. (B) HeLa cells were transiently transfected with plasmids encoding SPG21-mCherry, SPG21-T201Nfs-mCherry, SPG21-A108P-mCherry, SPG21-S109A-mCherry, SPG21-ΔN-mCherry (N-terminal helix deletion), or SPG21-ΔC-mCherry (C-terminal helix deletion). Cell extracts were analyzed by SDS–PAGE and immunoblotting with antibodies to mCherry and GAPDH. Positions of molecular mass markers (in kDa) are indicated. (C) WT HeLa cells were transiently transfected with the same plasmids as in panel B, stained with LysoTracker-Green, and imaged live by confocal fluorescence microscopy; scale bars, 10 µm. (D) Box-and-whisker plot of the Pearson's correlation coefficient for SPG21-mCherry and LysoTracker-Green from images such as those in panel C. Each data point represents one cell. Statistical significance was calculated by one-way ANOVA with multiple comparisons using the Dunnett's multiple comparisons test. ****P < 0.0001.

Next, we examined the effect of *SPG21* mutations on the expression and localization of SPG21-mCherry in live HeLa cells (Figure 4). We performed site-directed mutagenesis of SPG21-mCherry to introduce the Old Order Amish variant T201Nfs that induces a frameshift and premature stop codon (Cross and McKusick, 1967; Simpson *et al.*, 2003), or the Japanese missense variant A108P (Ishiura *et al.*, 2016), both within the α/β hydrolase domain of SPG21 (Figure 4A). We also mutated S109 to alanine because this residue was previously reported to be required for the interaction of SPG21 with CD4 (Zeitlmann *et al.*, 2001). Finally, we introduced mutations that delete the N-terminal or C-terminal α-helixes of SPG21 (ΔN and ΔC, respectively) (Figures 1A and 4A).

HeLa cells were transfected with equal amounts of plasmid DNA encoding WT and mutant SPG21-mCherry, and cell extracts were immunoblotted with an antibody to mCherry to examine total levels of SPG21-mCherry in each condition (Figure 4B). We observed that both the T201Nfs and A108P patient mutations, as well as the N-terminal α-helix deletion, greatly decreased the expression levels of SPG21-mCherry. The C-terminal α-helix deletion also decreased the expression levels of SPG21-mCherry, albeit to a lesser extent. In contrast, the S109A mutant was expressed at WT levels.

Analysis of the localization of the mutant SPG21-mCherry constructs showed that the small amounts of the T201Nfs, A108P and ΔN constructs expressed in these cells were completely cytosolic (Figure 4, C and D). In contrast, the ΔC and S109A constructs localized to endolysosomes, as observed by costaining with LysoTracker-Green (Figure 4, C and D).

From these experiments, we concluded that the T201Nfs and A108P patient variants are unstable and unable to associate with endolysosomes, and that the S109 residue involved in interaction with CD4 (Zeitlmann *et al.*, 2001) is dispensable for endolysosomal association of SPG21. Furthermore, the N-terminal helix, but not the C-terminal α-helix, is required for recruitment to endolysosomes. Because the N-terminal α-helix is likely not involved in interaction with RAB7A (Figure 3F), its requirement for association of SPG21 with endolysosomes may reflect an additional role in membrane targeting or in protein folding, as suggested by the low expression level of the ΔN mutant (Figure 4B).

### SPG21 and RAB7A colocalize in primary hippocampal neurons

**FIGURE 5:**
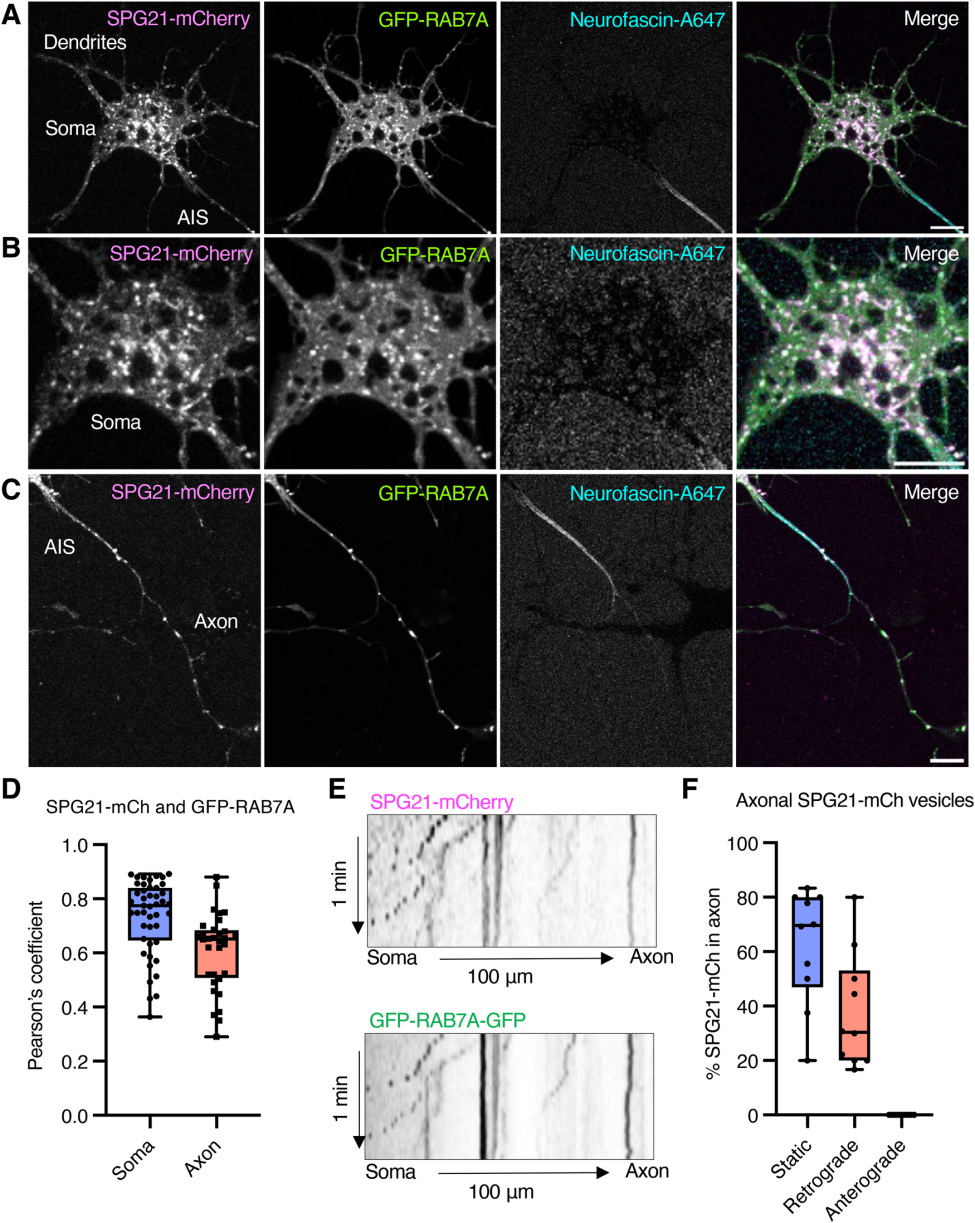
Colocalization of SPG21 and RAB7A in neurons. (A–C) DIV4 rat primary hippocampal neurons in primary culture were transiently transfected with plasmids encoding SPG21-mCherry (magenta) and GFP-RAB7A (green), labeled with Alexa Fluor 647-conjugated antibody to neurofascin (neurofascin-A647) (blue) (to label the AIS), and imaged live by confocal fluorescence microscopy at DIV5. Panel A shows the soma, dendrites and beginning of the AIS. Panel B shows an enlarged view of the soma. Panel C shows an enlarged view of the AIS and proximal axon; scale bars, 10 µm. (D) Box-and-whisker plot of the Pearson's correlation coefficient for SPG21-mCherry and GFP-RAB7A in the soma and axon. Each data point represents one cell. (E) Kymographs of SPG21-mCherry and GFP-RAB7A from a video of 100 µm of axon, with each frame taken every 3 s for a total of 1 min. (F) Box-and-whisker plots of the percentage of stationary, retrograde, and anterograde SPG21-mCherry–positive endolysosomes in 100 µm of axon calculated from kymographs generated from 10 different cells.

Because mutations in SPG21 lead to neurological deficits, we examined the localization of SPG21-mCherry in rat hippocampal neurons by live-cell imaging. To this end, SPG21-mCherry and GFP-RAB7A were expressed by transfection into day-in-vitro 5 (DIV5) rat hippocampal neurons in primary culture. The axon was identified by staining of the axon initial segment (AIS) with an Alexa Fluor 647–conjugated antibody to neurofascin (Farías *et al.*, 2015). We observed robust colocalization between SPG21-mCherry and GFP-RAB7A on vesicles throughout the soma, dendrites, AIS, and axon (Figure 5, A–D). The T201Nfs and A108P patient mutations abrogated the association of SPG21-mCherry with LysoTracker-Green-positive endolysosomes in neurons (Supplemental Figure S3) as they did in HeLa cells (Figure 4, C and D). Time-lapse imaging of the proximal axon (∼100 µm from the soma) by spinning-disk confocal microscopy showed the presence of colocalized SPG21-mCherry and GFP-RAB7A in both static and retrograde-moving, but not anterograde-moving, vesicles (Figure 5, E and F). This finding is consistent with the predominant association of RAB7A with axonal retrograde vesicles (Deinhardt *et al.*, 2006).

### SPG21 promotes noncanonical mTORC1-catalyzed phosphorylation of TFEB

**FIGURE 6:**
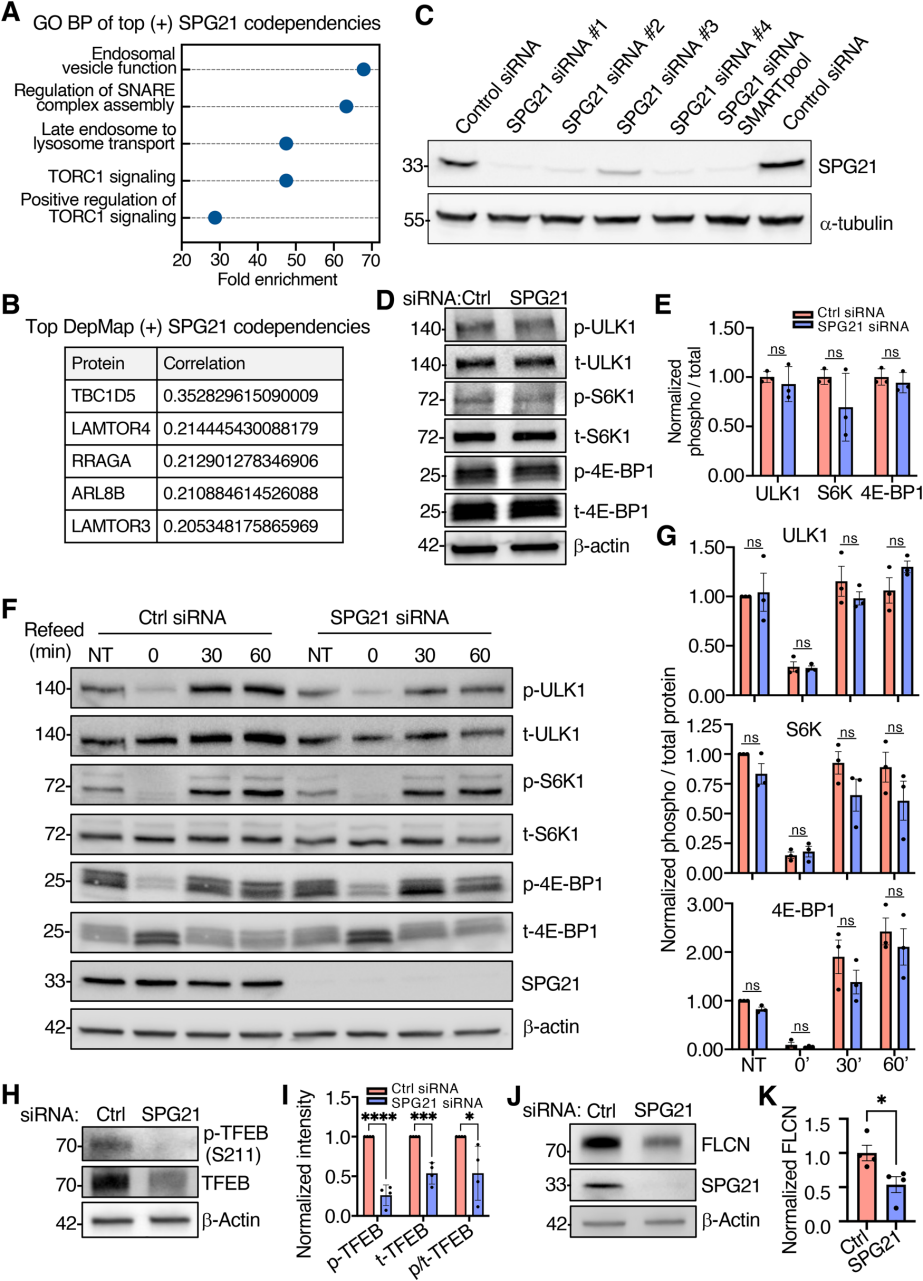
SPG21 depletion decreases mTORC1-catalyzed phosphorylation of TFEB at residue S211. (A) The codependency of SPG21 and other genes was analyzed using DepMap (https://depmap.org/portal/gene/SPG21?tab = overview). Of the top 100 codependencies with SPG21 from the DepMap CRISPR dataset (DepMap Public 25Q2+Score, Chronos), 64 showed a positive correlation and 36 a negative correlation. The top 64 positively correlated genes were used to perform a GO BP enrichment analysis. (B) Top five genes with the highest positive correlation with SPG21 in DepMap CRISPR dataset. (C) Silencing of SPG21 in HeLa cells was performed by transfection for 72 h with a pool of four siRNAs (i.e., SMARTpool) or each of the four siRNAs separately. Cells were analyzed by immunoblotting with antibodies to SPG21 or α-Tubulin (control). Subsequent experiments were done using SPG21 siRNA #1. (D) SDS–PAGE and immunoblot analysis of mTORC1 kinase activity in Hela cells treated for 72 h with a control (Ctrl) or SPG21 siRNA using antibodies to the phosphorylated (p) and total (t) forms of the indicated canonical substrates, and to β-actin as a loading control. (E) Quantification of mTORC1 canonical substrate phosphorylation from three independent experiments like that shown in panel D. (F) SDS–PAGE and immunoblot analysis of mTORC1 kinase activity in response to starvation (1 h, “0 min”) and refeeding (30 and 60 min) in HeLa cells treated for 72 h with a control (Ctrl) or SPG21 siRNA using antibodies to the phosphorylated (p) and total (t) forms of the indicated canonical substrates, and β-actin. (G) Quantification of mTORC1 canonical substrate phosphorylation in response to starvation and refeeding from three independent experiments like that shown in panel F. (H) SDS–PAGE and immunoblot analysis of S211-phosphorylated TFEB and total TFEB in HeLa cells treated for 72 h with control of SPG21 siRNA. β-actin was used as a loading control. (I) Quantification of phosphorylated TFEB (S211), total TFEB, and ratio of phosphorylated to total TFEB from four independent experiments like that shown in panel H. (J) HeLa cells transfected with control (Ctrl) and SPG21 siRNAs for 72 h, analyzed by SDS–PAGE, and immunoblotted with antibodies to folliculin (FCLN), SPG21 and β-actin. (K) Quantification of folliculin levels from four independent experiments like that shown in panel J. In panels C, D, F, H, and J, positions of molecular mass markers (in kDa) are indicated on the left. In panels E, G, I, and K, data are represented as the mean ± SEM. Statistical significance for the indicated comparisons was analyzed using a two-tailed unpaired Student's *t* test (panels E, I, and K) or two-way ANOVA with Tukey's multiple comparison test (panel G). ****P < 0.0001, ***P <0.001, *P < 0.05, ns, not significant.

To date, SPG21 has not been functionally linked to any endolysosomal process. To explore potential functions of SPG21, we analyzed SPG21 gene codependency patterns on DepMap (https://depmap.org/portal/). A positive correlation in this analysis suggests involvement of proteins in the same cellular pathways supporting viability of cancer cells (McFarland *et al.*, 2018). Gene ontology biological processes (GO BP) analysis (https://geneontology.org) of the top 64 positively correlated genes revealed an enrichment of SPG21 with endolysosomal functions and mTORC1 signaling (Figure 6A). The top five positively correlated genes encoded the RAB7A GTPase-activating protein (GAP) TBC1D5 (Seaman *et al.*, 2018), the mTORC1 signaling regulators LAMTOR4, LAMTOR3 (components of the Ragulator complex) and RRAGA (RagA) (Sancak *et al.*, 2010), and the small GTPase ARL8B (Hofmann and Munro, 2006) (Figure 6B)—all associated with endolysosomal compartments. This suggested that SPG21 not only localizes to endolysosomes, but may also function in association with these organelles.

We were particularly intrigued by the strong correlation between SPG21 and mTORC1 signaling suggested by DepMap. mTORC1 is a serine/threonine kinase complex that phosphorylates canonical substrates such as ULK1, S6K1, and 4E-BP1 in response to nutrient availability, energy status, and growth factors (Condon and Sabatini, 2019; Goul *et al.*, 2023). This activity is regulated by a signaling axis involving the Ragulator complex (LAMTOR1–5), the heterodimeric Rag small GTPases (RRAGA/RRAGB–RRAGC/RRAGD), and the small GTPase RHEB, which together coordinate mTORC1 activation at the endolysosomal membrane (Condon and Sabatini, 2019; Goul *et al.*, 2023). To investigate a potential role for SPG21 in mTORC1 signaling, we used siRNA-mediated KD to deplete SPG21 in HeLa cells. KD was favored over KO to minimize the chance of adaptation or compensation (El-Brolosy and Stainier, 2017) in response to loss of SPG21. Efficient (>90%) SPG21 depletion was achieved by treating cells for 72 h with either an siRNA pool or any three of the four siRNAs in the pool (Figure 6C). Subsequent experiments were done using siRNA #1. SPG21-depleted cells exhibited normal appearance of endolysosomes stained for endogenous LAMP1 (Supplemental Figure S4A), as well as unaltered processing of the endolysosomal acid hydrolase cathepsin D (Supplemental Figure S4B). LC3B staining, levels, processing, and lysosomal delivery were also unaffected by KD of SPG21 (Supplemental Figure S4, C–E). These findings suggested that SPG21 KD does not cause global alterations in endolysosomal and autophagy function under the conditions of our experiments. Surprisingly, despite the potential connection of SPG21 to mTORC1 signaling (Figure 6, A and B), SPG21 KD did not affect mTORC1-catalyzed phosphorylation or total levels of ULK1, S6K1, and 4E-BP1 under basal conditions (Figure 6, D and E), or upon starvation and refeeding (Figure 6, F and G).

Could SPG21 and mTORC1 still be functionally connected? In addition to phosphorylating canonical substrates, mTORC1 has been shown to phosphorylate the bHLH transcription factor TFEB via a noncanonical pathway. In this pathway, TFEB is recruited to the endolysosomal surface by the GDP-bound form of the RRAGC/RRAGD GTPase (Martina and Puertollano, 2013; Napolitano *et al.*, 2020; Cui *et al.*, 2023; reviewed by Napolitano *et al.*, 2022). Indeed, we observed that SPG21 KD led to a marked reduction in phosphorylated TFEB and, to a lesser extent, total TFEB (Figure 6, H and I). This differential effect resulted in a decreased ratio of phosphorylated to total TFEB in SPG21-KD cells compared with control cells (Figure 6I). Because folliculin (FCLN) acts as GAP to promote conversion of RRAGC/RRAGD to the GDP-bound form in the noncanonical pathway (Petit *et al.*, 2013; Tsun *et al.*, 2013; Napolitano *et al.*, 2020; Alesi *et al.*, 2021; Napolitano *et al.*, 2022), we compared the levels of FLCN in SPG21-KD and control cells. We observed that SPG21 depletion decreased the total levels of FLCN, potentially contributing to the decrease in noncanonical mTORC1-catalyzed phosphorylation of TFEB (Figure 6, J and K).

From these analyses, we concluded that SPG21 promotes mTORC1-catalyzed phosphorylation of the noncanonical substrate TFEB, but not the canonical substrates ULK1, S6K1, and 4E-BP1.

### SPG21 promotes TFEB cytoplasmic localization and inactivation

TFEB is a transcription factor that regulates the expression of numerous lysosomal and autophagy-related genes (Sardiello *et al.*, 2009; Settembre *et al.*, 2011). Its activity is controlled by phosphorylation-dependent translocation between the cytoplasm and the nucleus (Settembre *et al.*, 2011). mTORC1-catalyzed phosphorylation keeps TFEB in the cytoplasm, while dephosphorylation enables TFEB transport into the nucleus to regulate transcription (Peña-Llopis *et al.*, 2011; Settembre *et al.*, 2012; Martina *et al.*, 2012; Roczniak-Ferguson *et al.*, 2012).

**FIGURE 7:**
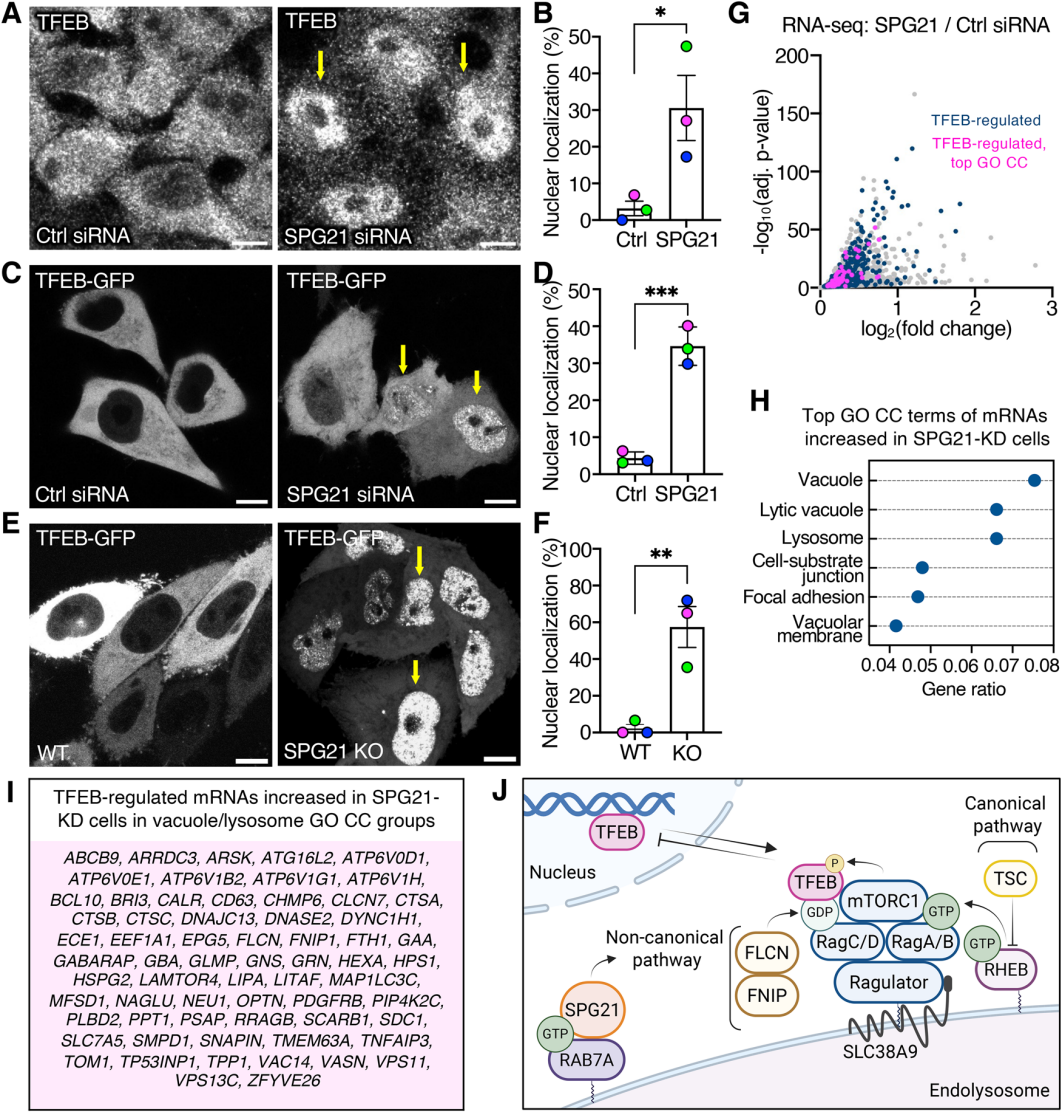
SPG21 depletion causes TFEB redistribution to the nucleus and increased transcription of TFEB-regulated genes. (A) Immunofluorescence microscopy of HeLa cells treated with control or SPG21 siRNA, fixed, permeabilized and immunostained for endogenous TFEB; scale bars, 10 µm. (B) Quantification of TFEB nuclear:cytoplasmic ratio from three independent experiments like that shown in panel A. (C) Fluorescence microscopy of HeLa cells treated with control or SPG21 siRNA and transiently transfected with plasmid encoding TFEB-GFP. (D) Quantification of TFEB-GFP nuclear:cytoplasmic ratio from three independent experiments like that shown in panel C. (E) Fluorescence microscopy of WT and SPG21-KO HeLa cells transiently transfected with a plasmid encoding TFEB-GFP. In A, C and E, arrows point to TFEB-GFP in the nucleus. (F) Quantification of TFEB-GFP nuclear:cytoplasmic ratio from three independent experiments like that shown in panel E. In B, D and F, data are represented as the mean ± SEM. The statistical significance was calculated using a two-tailed unpaired Student's *t* test. ***P < 0.001, **P < 0.01, *P < 0.05. (G) Volcano plot of upregulated mRNAs in RNA-Seq of HeLa cells treated with SPG21 siRNA relative to control siRNA. Complete RNA-Seq data are shown in Supplemental Table S1. Abundance is quantified as log_2_(fold change) on the *x*-axis and significance as −log_10_(adjusted *P* value) on the *y*-axis. mRNAs were considered upregulated if they had a greater than 1.05-fold increase and less than 0.05 adjusted *P* value. TFEB-upregulated mRNAs represented in the vacuole/lytic vacuole/lysosome/vacuolar membrane terms in panel H (65) are highlighted in magenta, and other TFEB-upregulated mRNAs (615) are highlighted in dark blue. (H) Dotplot of top six terms identified in GO CC analysis of mRNAs upregulated in SPG21-KD HeLa cells. (I) Specific mRNAs in the vacuole/lytic vacuole/lysosome/vacuolar membrane terms in panel H that are upregulated in SPG21-KD cells and are known TFEB-regulated genes. (J) Schematic representation of SPG21 as a RAB7A effector that promotes FLCN-mediated, mTORC1-catalyzed noncanonical phosphorylation of TFEB, suppressing TFEB nuclear translocation and gene expression.

To determine whether SPG21-dependent phosphorylation of TFEB by mTORC1 regulates mTORC1 nuclear-cytoplasmic shuttling, we examined the localization of endogenous TFEB and transgenic TFEB-GFP in control and SPG21-KD cells. We observed that SPG21 KD led to nuclear localization of TFEB from virtually 0 to ∼30% of the cells (Figure 7, A–D). The nuclear redistribution of TFEB-GFP was even more pronounced in SPG21-KO cells (∼60%) (Figure 7, E and F). This redistribution was consistent with the reduced fraction of phosphorylated TFEB (Figure 6, H and I).

The redistribution of TFEB to the nucleus in SPG21-depleted cells would be expected to enhance the transcription of TFEB-regulated genes. Indeed, RNA sequencing showed that SPG21 KD caused a significant increase (at least 1.05-fold change and less than 0.05 adjusted *P* value) in 680 mRNAs previously demonstrated to be regulated by TFEB (Figure 7G, dark blue and magenta dots; Supplemental Table S1). Four of the top six terms in GO cellular component (CC) analysis of increased mRNAs corresponded to the vacuole/lysosome (Figure 7H). This vacuolar/lysosomal subset itself included 65 mRNAs previously demonstrated to be regulated by TFEB (Figure 7I; magenta dots). Notably, the TFEB mRNA itself was slightly but significantly downregulated in the RNA sequencing analysis (KD/WT fold change = 0.821; *P*_adj_ value = 0.000466393; Supplemental Table S1). This could explain the decreased levels of total TFEB protein in SPG21-KD cells (Figure 6, H and I), although we cannot rule out that this decrease could be due to increased degradation after activation, as previously suggested by other studies (Yang *et al.*, 2024; Tapia *et al.*, 2025).

Overall, these results demonstrate that SPG21 promotes TFEB phosphorylation and cytoplasmic retention, thereby reducing the transcription of a subset of TFEB target genes.

## DISCUSSION

Despite significant progress in the identification of genes that are mutated in different types of HSP, the localization and function of many of the corresponding proteins remain poorly defined (Blackstone, 2018; Elsayed *et al.*, 2021). A notable example is the Mast syndrome protein SPG21, which was previously found to exhibit a diffuse cytoplasmic distribution, with some perinuclear clusters that roughly colocalized with endosomal and Golgi/TGN markers by immunofluorescence microscopy (Zeitlmann *et al.*, 2001; Hanna and Blackstone, 2009). The predominance of the diffuse pattern, however, made it difficult to ascertain if the localization with marker proteins reflected true association with specific organelles. Our initial efforts to determine a more precise localization of either endogenous SPG21 or transgenic SPG21-GFP by immunofluorescence microscopy were similarly hindered by the diffuse and nondescript cytoplasmic distribution observed for both forms (Figure 1, B and C). However, our live-cell imaging of SPG21-GFP or SPG21-mCherry in HeLa cells revealed a clear localization to discrete vesicles colabeled with the fluorescently tagged endolysosomal markers LAMP1, RAB7A, ARL8B and RAB9A, and with the acidophilic, fluorogenic dye LysoTracker (Figures 1, D–G and 2, A and B). In contrast, there was little or no colocalization of SPG21-mCherry with early endosomal and TGN markers (Figure 2B). We also observed colocalization of SPG21-mCherry with GFP-RAB7A in live rat hippocampal neurons, including on endolysosomal structures in the soma and dendrites, and on retrograde transport carriers in the axon (Figure 5). These findings demonstrated that SPG21 specifically associates with endolysosomal organelles (i.e., late endosomes, lysosomes, and related vesicles) in both nonneuronal cells and neurons. FRAP experiments showed that the association of SPG21-GFP with endolysosomes is dynamic, rapidly cycling between membrane-bound and cytosolic pools (Figure 1, F–H). The lability of SPG21 association with membranes likely explains its disruption during the fixation and permeabilization steps of immunofluorescence microscopy.

The ability to visualize the localization of fluorescently labeled SPG21 in living cells allowed us to investigate how this protein is recruited to endolysosomes. Live-cell imaging of cells with KO or KD of several endolysosomal small GTPases revealed that RAB7A is required for the association of SPG21 with endolysosomes (Figure 3, A–C). Moreover, coimmunoprecipitation analyses demonstrated a GTP-dependent interaction of GFP-RAB7A with endogenous SPG21 (Figure 3, D and E). These results are consistent with SPG21 being a RAB7A effector, like others that regulate late endosomal and autophagic processes (Langemeyer *et al.*, 2018; Mulligan and Winckler, 2023). Most cell types express a RAB7B paralogue that exhibits 50% amino-acid identity to RAB7A (Yang *et al.*, 2004). However, this protein is not directly involved in late endocytic/autophagic processes and instead mediates transport between endosomes and the TGN (Progida *et al.*, 2010; Progida *et al.*, 2012).

Being able to observe the endolysosomal localization of fluorescently tagged SPG21 in live cells also allowed us to examine the effect of SPG21 patient mutations on the distribution of the protein. We investigated the T201Nfs variant (Cross and McKusick, 1967; Simpson *et al.*, 2003), which is the longest of the prematurely terminated proteins (Simpson *et al.*, 2003; Scarlato *et al.*, 2017), and A108P (Ishiura *et al.*, 2016), the only missense variant reported to date. Both mutations caused severe reductions in SPG21 levels and its association with endolysosomes (Figure 4). The presence of trace amounts of the A108P mutant may explain why this form of the disease presents later in life and with less severity (i.e., absence of cerebellar, extrapyramidal, or bulbar signs) (Ishiura *et al.*, 2016) than that caused by the T201Nfs and other truncated variants (Simpson *et al.*, 2003; Scarlato *et al.*, 2017; Amprosi *et al.*, 2022).

The endolysosomal localization of SPG21 prompted us to investigate its potential role at this location. Among all the processes examined, the regulation of TFEB phosphorylation by mTORC1 emerged as the most affected. mTORC1 phosphorylates substrates by two pathways referred to as canonical and noncanonical (Figure 7I). The canonical pathway involves sensing of nutrient levels by the SLC38A9 amino-acid transporter, followed by sequential activation of the Ragulator complex and exchange of GTP for GDP on RRAGA/RRAGB (Condon and Sabatini, 2019; Goul *et al.*, 2023). This leads to the recruitment of mTORC1 to the endolysosomal surface and its activation by the growth-factor–induced RHEB GTPase. mTORC1 then phosphorylates canonical substrates such as ULK1, S6K, and 4E-BP.

The noncanonical pathway shares the amino-acid–dependent mTORC1 recruitment via SLC38A9 and RRAGA-GTP, but does not require mTORC1 activation by RHEB (Napolitano *et al.*, 2020; Napolitano *et al.*, 2022). Instead, it involves the recruitment of its substrate TFEB to endolysosomes by the GDP-bound form of RRAGC (Martina and Puertollano, 2013; Napolitano *et al.*, 2020; Cui *et al.*, 2023) (Figure 7I). RRAGC-GDP is generated by the GAP activity of FLCN, which exists as a complex with FNIP1/2 (Tsun *et al.*, 2013; Napolitano *et al.*, 2020; Alesi *et al.*, 2021; Napolitano *et al.*, 2022).

Remarkably, we observed that SPG21 depletion decreased the ratio of mTORC1-phosphorylated to total TFEB (Figure 6, H and I), without affecting the phosphorylation of canonical mTORC1 substrates such as ULK1, S6K, and 4E-BP (Figure 6, D–G). Furthermore, SPG21 depletion decreased the levels of FLCN (Figure 6, J and K). These findings thus demonstrated the specific involvement of SPG21 in the noncanonical mTORC1 signaling pathway, possibly via changes in FLCN levels or FLCN sequestration in an inactive form (Goodwin *et al.*, 2021; Napolitano *et al.*, 2022; Jeong *et al.*, 2024). In this regard, FLCN levels and activity have been shown to be modulated by sequestration by membrane-conjugated GABARAP (Goodwin *et al.*, 2021), TMEM55B (Jeong *et al.*, 2024), and AMPK-dependent phosphorylation of FNIP1 (Malik *et al.*, 2023). At present, it remains unclear whether SPG21 is involved in this pathway directly or indirectly. In the latter case, the regulation of TFEB phosphorylation by SPG21 could be mediated through upstream regulators of FLCN or other alterations of endolysosomal homeostasis.

The decrease in TFEB phosphorylation (Figure 6, H and I) promotes its nuclear translocation and the induction of a subset of TFEB target genes (Figure 7, G and H). The observation that SPG21 loss does not activate the full repertoire of TFEB-regulated genes suggests a selective transcriptional response. Consistent with this limited activation, we did not detect global changes in lysosomal properties or autophagy over the time course of our experiments. SPG21 (Soderblom *et al.*, 2010; Davenport *et al.*, 2016), mTORC1 (Cloëtta *et al.*, 2013; Andrews *et al.*, 2020), and TFEB (Meireles *et al.*, 2018; Sun *et al.*, 2018) have all been implicated in neurodevelopment. It is likely that loss of SPG21-dependent regulation of mTORC1 and TFEB alters neuronal and/or glial development, contributing to the pathogenesis of SPG21-linked HSP.

SPG21 thus joins the group of HSP gene products that are associated with endolysosomal compartments, including SPG11, SPG15, SPG20, AP-5, NIPA1, WASHC5, VPS37A, and ATP13A2 (Blackstone, 2018; Elsayed *et al.*, 2021). The prevalence of this gene set among all causes of HPS emphasizes the critical importance of endolysosomes in CNS development and function.

## MATERIALS AND METHODS

Request a protocol through *Bio-protocol*

### Antibodies and fluorescent probes

We used primary antibodies to the following proteins: SPG21 (rabbit polyclonal, Sigma-Aldrich, HPA040407, 1:1000), LAMP1 (mouse monoclonal, DSHB Hybridoma Product H4A3, deposited by J.T. August and J.E.K. Hildreth, 1:400), horseradish peroxidase (HRP)-conjugated anti-GFP (MACS, 130091833, 1:2000), mCherry (rat monoclonal, Invitrogen, 16D7, 1:1000), HRP-conjugated GAPDH (mouse monoclonal, Santa Cruz Biotechnology, sc-47724 HRP, 1:1000), RAB7A (rabbit monoclonal, Cell Signaling Technology, D95F2, 1:100), HRP-conjugated α-Tubulin (mouse monoclonal, Santa Cruz Biotechnology, sc-5286 HRP, 1:1000), RagB (rabbit monoclonal, Cell Signaling Technology, D18F3, 1:1000), LC3B (rabbit monoclonal, Cell Signaling Technology, 3868, 1:200), Cathepsin D (rabbit polyclonal, Cell Signaling Technology, 2284s, 1:1000), TFEB (rabbit polyclonal, Cell Signaling Technology, 4240, 1:1000), p-TFEB (Ser211) (rabbit monoclonal, Cell Signaling Technology, 37861, 1:1000, gifted by Shu Yang, NICHD, NIH), S6K (rabbit monoclonal, Cell Signaling Technology, 2708, 1:1000), p-S6K (rabbit monoclonal, Cell Signaling Technology, 9234, 1:1000), ULK1 (rabbit monoclonal, Cell Signaling Technology, 8054, 1:1000), p-ULK1 (757) (rabbit monoclonal, Cell Signaling Technology, 14202, 1:1000), 4E-BP1 (rabbit monoclonal, Cell Signaling Technology, 9644, 1:1000), p-4E-BP1 (rabbit monoclonal, Cell Signaling Technology, 2855, 1:1000), FLCN (rabbit monoclonal, Cell Signaling Technology, 3697, 1:100), and β-actin-HRP (mouse monoclonal, Sigma Aldrich, A3854, 1:2000). In addition, we used the following secondary antibodies: Alexa Fluor 488–conjugated donkey anti-rabbit IgG (Invitrogen, A21206, 1:1000), Alexa Fluor 488–conjugated donkey anti-mouse IgG (Invitrogen, A21202, 1:1000), Alexa Fluor 555–conjugated donkey anti-rabbit IgG (Invitrogen, A31572, 1:1000), Alexa Fluor 555–conjugated donkey anti-mouse IgG (Invitrogen, A31570, 1:1000), and HRP-conjugated donkey anti-rabbit IgG (GE Healthcare, NA934V, 1:5000). We also used the following fluorescent probes: LysoTracker Red DND-99 (Invitrogen, L7528, used at 50 nM) and LysoTracker Green DND-26 (Invitrogen, L7526, used at 50 nM). We labeled Anti-Pan-Neurofascin (extracellular) antibody (A12/18) (Antibodies, Inc., 74-172) with the Mix-n-Stain CF640R Antibody-Labeling Kit (Biotium, 92245), according to the manufacturer's protocol.

### Plasmids and cell lines

Complementary DNA (cDNA) encoding human SPG21 was cloned into pEGFP-C1 (Clontech). SPG21 cDNA was also cloned into pmCherry-N1 (Clontech). Site-directed mutagenesis of SPG21 plasmids (ΔN, ΔC, A108P, S109A, and T201n fs) was done by using the Q5 Site-Directed Mutagenesis Kit (New England Biolabs, E0554S). The sequences of all constructs were confirmed by Sanger DNA sequencing (GeneWiz/Azenta). Plasmids encoding ARL1-GFP (Ishida and Bonifacino, 2019), ARL8B-mCherry and ARL8B-GFP (Farías *et al.*, 2017), GFP-RAB7A, GFP-RAB7A-T22N, and GFP-RAB7A-Q67L (Rojas *et al.*, 2008) were described previously. A plasmid encoding TFEB-GFP was a gift from Carlos Guardia (NIEHS, NIH).

### Cell culture, transfection, and KDs

Human HeLa (catalogue no. CCL-2, ATCC), HEK293T (catalogue no. 632180, Takara Bio), H4 (catalogue no. HTB-148, ATCC), RAB9A-KO HeLa (Keren-Kaplan and Bonifacino, 2021), and RAB7A-KO HeLa cells (gift from Morié Ishida, NICHD, NIH) were grown in DMEM (catalogue no. 112-319-101, Quality Biological) supplemented with 10% FBS (35-011-CV, Corning), 100 IU/ml penicillin, 100 µg/ml streptomycin (catalogue no. 30002-CL, Corning), and 2 mM l-glutamine (referred to as complete medium or CM) at 37°C and 5% CO_2_. ARL8A-B-KO HeLa cells were described previously (Keren-Kaplan and Bonifacino, 2021).

Primary rat (E18) hippocampal neurons were prepared as described previously (Farías *et al.*, 2016), plated on cover glasses coated with poly-lysine (Sigma) and laminin (Roche), and grown in Neurobasal medium (Life Technologies) supplemented with B27 (Thermo Fisher Scientific). Neurons were transfected on day-in-vitro 4 (DIV4) and imaged on DIV5.

Lipofectamine 2000 (catalogue no. 11668019, Thermo Fisher Scientific) was used for transfections according to the manufacturer's protocol. Cells were fixed or imaged live ∼24 h after transfection. For coimmunoprecipitation experiments, 5 to 7 µg plasmid DNA and 8 µl Lipofectamine were used per 10-cm plate. Cells were harvested ∼24 h after transfection.

For KD, HeLa or H4 cells were plated on cover glasses or plates and transfected with siRNAs: ON-TARGETplus nontargeting control pool siRNA (Dharmacon, D-001810-10-05); SPG21 ON-TARGETplus Human siRNA (Dharmacon, siRNA #1: J-013476-09-0002, siRNA #2: J-013476-10-0002, siRNA #3: J-013476-11-0002, siRNA #4: J-013476-12-0002, SMARTpool: L-013476-01-0005); RagB ON-TARGETplus Human siRNA (Dharmacon, L-012189-01-0005) (gifted by Shu Yang, NIH) using the Lipofectamine RNAiMAX Transfection Reagent (Invitrogen, 13778075). After 72 h, cells were fixed, imaged live, or lysed for immunoblotting.

### Immunofluorescence microscopy

Cells were fixed with 4% paraformaldehyde (PFA) for 20 min at room temperature or with –20°C methanol for 5 min, washed twice with PBS, incubated with PBS supplemented with 0.1% saponin and 0.5–1% BSA (blocking buffer) for 30 min at room temperature, incubated with primary antibodies diluted in blocking buffer for 1 h at room temperature, washed five times with PBS, incubated with secondary antibodies diluted in blocking buffer for 1 h at room temperature, washed five times with PBS and twice with distilled water, and mounted on slides using Fluoromount-G with DAPI (catalogue no. 0100-20, Electron Microscopy Sciences).

### Image acquisition

Images were acquired on a Zeiss LSM880 inverted confocal laser scanning microscope fitted with a Plan-Apochromat 63X, 1.4 numerical aperture (NA) objective (Carl Zeiss). Live-cell imaging was performed in a controlled chamber (37°C and 5% CO_2_). Z-stacks were obtained, and maximal intensity projections were generated. Microcopy images were acquired with Zeiss ZEN Black software: Zen 2012 SPF FP3 release version 14.0.22.201, and Zen 2.3 SP1 FP3 release version 14.0.25.201. Images were further processed in Fiji (Schindelin *et al.*, 2012).

### FRAP analysis

We performed fluorescence recovery after photobleaching (FRAP) in live HeLa cells expressing SPG21-GFP incubated at 37°C and 5% CO_2_. A region of interest (endolysosomal SPG21-GFP) was photobleached, using the bleaching pulse in the Zeiss Zen Black software. Following photobleaching, images were taken at one frame per second for 60 s. Fluorescence intensities in the regions of interest were corrected for background fluorescence and normalized to background (control region) to account for scan-related bleaching. We quantified these traces using a Jython script for FRAP analysis (https://imagej.net/tutorials/analyze-frap-movies) (Schindelin *et al.*, 2012). Nonlinear regressions were modeled using a One-phase association in GraphPad Prism (Figure 1H)

### SDS–PAGE and immunoblotting

Samples were lysed in Laemmli sample buffer containing β-mercaptoethanol for 5 min at 95°C, resolved by SDS–PAGE and transferred onto a nitrocellulose membrane (Bio-Rad). Membranes were blocked with 5% blotting-grade milk (Bio-Rad) or 5% BSA (GoldBio) in TBS containing 0.1% Tween 20 (Sigma-Aldrich) for 20 min at room temperature and incubated with the indicated primary antibodies for 1 h at room temperature. HRP-conjugated secondary antibodies were incubated with the membrane at room temperature for 1 h. HRP was detected using SuperSignal West Dura Extended Duration Substrate (Thermo Fisher Scientific, 34075) or SuperSignal West Femto Maximum Sensitivity Substrate (Thermo Fisher Scientific, 34095). Images were captured using a ChemiDoc Imaging System (Bio-Rad).

### Coimmunoprecipitation

HEK293T cells (2.5 × 10^6^) were plated on 10-cm dishes and transfected the following day. Cells were scraped and washed twice in cold PBS for 3 min at 4°C with a 500 × *g* spin between washes. Cell pellets were resuspended in 1 mL cold lysis buffer and incubated for 30 min at 4°C with gentle rotation. Lysis buffer composition was 10 mM Tris/Cl pH 7.4, 150 mM NaCl, 0.5 mM EDTA, 0.5% Nonidet P-40 Substitute (Proteintech, ChromoTek), supplemented with complete EDTA-free protease inhibitor tablet (catalogue no. 1836170, Roche). Following lysis, the soluble fraction was separated by centrifugation for 10 min at 4°C, 17,000 × *g*. Lysates were incubated with 25 µl GFP-TRAP Magnetic Agarose suspension (Proteintech, ChromoTek) for 1 h at 4°C with gentle rotation. Following incubation, beads were washed twice in 500 µl of 10 mM Tris-HCl pH 7.5, 150 mM NaCl, 0.05% Nonidet P-40 Substitute, 0.5 mM EDTA (Proteintech, ChromoTek), at room temperature. Washed beads were eluted by addition of Laemmli sample buffer containing β-mercaptoethanol, and heated for 5 min at 95°C. Samples were resolved by SDS–PAGE and immunoblotted.

### Assays of mTORC1 activity

HeLa cells were treated with control or SPG21 siRNAs for 72 h. Medium was replaced with fresh DMEM containing 10% FBS for a 30 min preincubation period. To perform serum starvation, cells were incubated with Earle's Balanced Salt Solution for 1 h. Following starvation, cells were refed by replacing the medium with fresh DMEM plus 10% FBS for 0, 30, or 60 min, or no treatment. Cells were lysed in Laemmli sample buffer containing β-mercaptoethanol and heated for 5 min at 95°C. Samples were resolved by SDS–PAGE and immunoblotted for canonical and noncanonical mTORC1 substrates.

### FACS-based autophagy assay

Delivery of LC3B from neutral autophagosomes to acidic lysosomes was analyzed using a FACS-based assay described by Jia and Bonifacino, 2019. Briefly, H4 cells expressing endogenously tagged GFP-mCherry-LC3B (H4-tfLC3B) were untreated or treated with control or SPG21 siRNA for 72 h before harvesting. One sample was treated with 100 nM bafilomycin A1 for 2 h before harvesting as a positive control. Cells were detached with 0.5% trypsin and 5 mM EDTA, neutralized with fresh DMEM plus 10% FBS (supplemented with 100 nM Bafilomycin A1 for its respective sample), pelleted, and fixed with 4% PFA. Fluorescence intensities were measured on an LSRFortessa Flow Cytometer (BD Biosciences). The GFP to mCherry ratio is inversely correlated to the integrity of lysosomal delivery of LC3B. Average GFP and mCherry signal was calculated from between 53,268 and 61,315 cells per sample, *n* = 3 per condition.

### RNA preparation and sequencing

HeLa cells cultured on 6-well plates were transfected with control and SPG21 siRNAs (four replicates each). At 72 h after transfection, total RNA from the KD cells was isolated with RNeasy Mini Kit (Qiagen), according to the manufacturer's protocol for purification of total RNA from human cells. RNA sequencing was performed by the NICHD Molecular Genetics Core as described previously (De Pace *et al.*, 2024).

### Bioinformatics analysis

A total of 100-bp paired-end reads for all samples (four replicates SPG21 KD, four replicates control KD) were trimmed with cutadapt v4.4 using arguments–a AGATCGGAAGAGCACACGTCTGAACTCCAGTCA-A AGATCGGAAGAGCGTCGTGTAGGGAAAGAGTGT–nextseq-trim 20–overlap 6–minimum-length = 25 to remove TruSeq adapters and perform light quality trimming. Trimmed reads were aligned to the GENCODE v28 human primary assembly using STAR v2.7.10b with parameters recommended by ENCODE for RNA-seq, specifically–outFilterType BySJout–outFilterMultimapNmax 20–alignSJoverhangMin 8–alignSJDBoverhangMin 1–outFilterMismatchNmax 999–outFilterMismatchNoverReadLmax 0.04–alignIntronMin 20–alignIntronMax 1000000–alignMatesGapMax 1000000–outSAMunmapped None.

Aligned reads were counted in genes using featureCounts v2.0.3 and the GENCODE v28 GTF primary assembly annotation. Extensive QC metrics showed good quality across all samples and treatment groups clustering as expected. The counts table was imported into R v4.2.3, and DESeq2 v 1.38.0 was used for differential expression with the model “∼group” to compare SPG21 KD with control and using the ashr log_2_FoldChange shrinkage method. Using a Benjamini–Hochberg corrected *P* value (false discovery rate) threshold of 0.05 and the null hypothesis that the log_2_FoldChange is different from zero, 3414 and 3762 genes were upregulated or downregulated, respectively.

Functional enrichment was performed with the clusterProfiler v4.6.0 enrichGO and enrichKEGG functions, using the GO database and ontologies BP, CC, and molecular function, and the KEGG database provided in the annotation (via AnnotationDbi, snapshot date 2024-11-13). The background for functional enrichment consisted of all genes that had nonzero expression in any sample.

To visualize upregulated TFEB-related transcripts, a list of TFEB responsive genes was generated by combining the genes reported on Harmonizome (https://maayanlab.cloud/Harmonizome/gene_set/TFEB/CHEA+Transcription+Factor+Targets) (Diamant *et al.*, 2025), with those reported in Supplemental Table S3 of Sardiello *et al.*, 2009, and in the TFEB_HeLa CHIP-seq dataset (https://tfeb.tigem.it/ChIP-seq_Palmieri_HMG.xlsx) (De Cegli *et al.*, 2022). Upregulated transcripts by RNA-Seq analysis that matched an identifier in this TFEB list were highlighted in Figure 7G and Supplemental Table S1.

### Quantification and statistics

Immunoblots were quantified with Fiji. Protein levels were first normalized to loading controls and then normalized by the average of control replicates for each experimental condition (Figures 3E and 6, E, G, I, and K). Colocalization analyses were performed using the Pearson–Spearman correlation plug-in for ImageJ/Fiji (Figures 4D and 5D). Kymographs were generated with Fiji (Figure 5E). Lines of one-pixel thickness and 50-µm length were tracked in the proximal axon and straightened, followed by stack reslicing and Z-projection. The percentage of SPG21-mCherry vesicles in the axon that were static, moving retrogradely, or anterogradely were quantified from the generated kymographs (Figure 5, E and F). Nuclear localization of endogenous TFEB and TFEB-GFP was analyzed by calculating the percentage of TFEB or TFEB-GFP–positive cells with nuclear expression (Figure 7, B, D, and F). Statistical analyses were performed using two-tailed unpaired Student's *t* test when two groups were compared (Figures 5, D, and F, 6, E, G, I, and K, and Figure 7, B, D, and J; Supplemental Figure S4E), one-way ANOVA when multiple groups were compared with Dunnett's multiple comparisons test (Figures 3E and 4D), and two-way ANOVA when multiple groups were compared across time and condition with Tukey's multiple comparison test (Figure 6G).

## Supporting information







## Data Availability

Reagents generated in this study are available upon request. RNA-Seq data were deposited at the Gene Expression Omnibus, accession number GSE304705 (https://www.ncbi.nlm.nih.gov/geo/query/acc.cgi?acc=GSE304705). All other data are available in the main text or the Supplementary Materials. Further information and requests for resources and reagents should be directed to the corresponding author.

## Abbreviations used:

AIS: axon initial segment
BP: biological process
CC: cellular component
CNS: central nervous system
DIV: day-in-vitro
ER: endoplasmic reticulum
FACS: fluorescence-activated cell sorting
FRAP: fluorescence recovery after photobleaching
GAP: GTPase-activating protein
GO: gene ontology
HSP: hereditary spastic paraplegia
KD: knockdown
KO: knockout
MRI: magnetic resonance imaging
TGN: *trans*-Golgi network
WT: wild-type.
