# Supplementary material for: The hereditary spastic paraplegia type 21 (SPG21) protein is a RAB7A effector that promotes noncanonical mTORC1-catalyzed TFEB phosphorylation and cytoplasmic retention
Source: Mol Biol Cell. 2025 Sep 12;36(10):ar123. doi: 10.1091/mbc.E25-07-0346 (PMC12483374; doi:10.1091/mbc.E25-07-0346)
Supplement: Supplementary file 1 [file mbc-36-ar123-s001.pdf]

Supplemental Materials

Molecular Biology of the Cell

Kunselman *et al*.

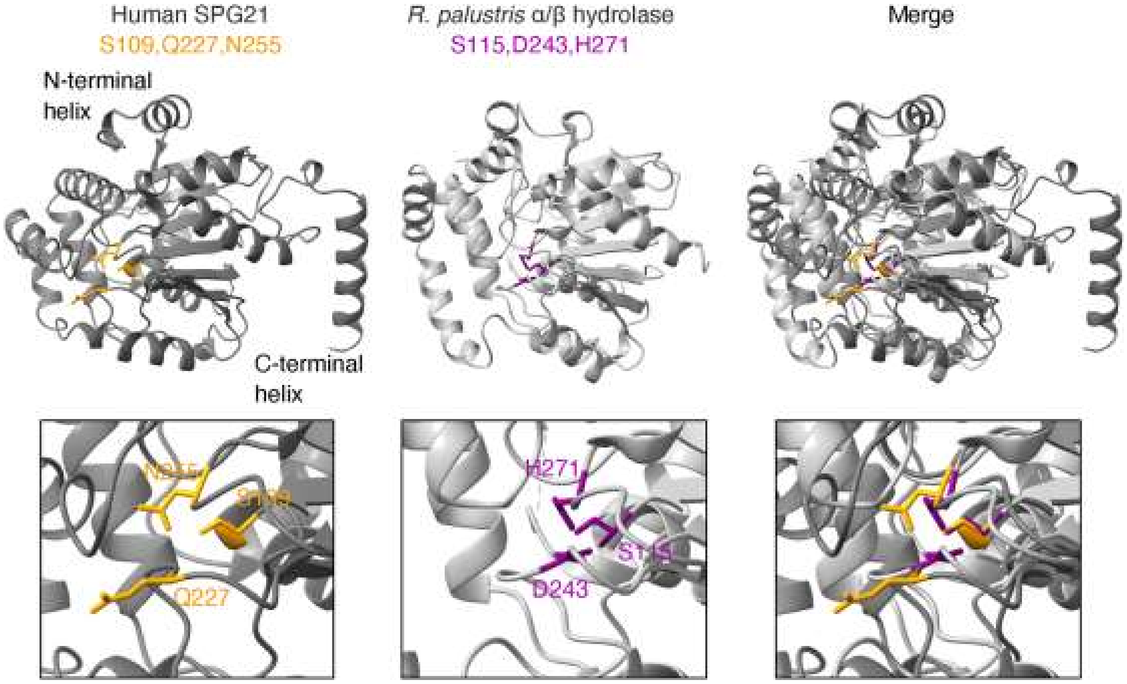

SUPPLEMENTAL FIGURE S1: Absence of conserved α/β hydrolase active site residues in SPG21. Predicted AlphaFold 3 structure (Abramson et al., 2024) of human SPG21 and crystal structure of *Rhodopseudomonas palustris* α/β hydrolase (PDB entry 4PSU). Highlighted are the catalytic triad in the α/β hydrolase and the matching residues in SPG21.

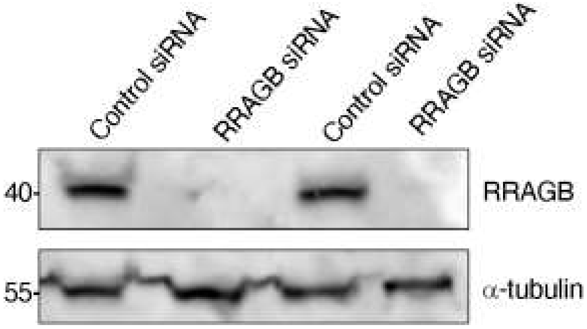

SUPPLEMENTAL FIGURE S2: Depletion of RRAGB by siRNA. HeLa cells were treated with control or RRAGB siRNAs for 48 h. Cell lysates were analyzed by SDS-PAGE and immunoblotting for endogenous RRAGB and α-tubulin (loading control). Representative image of two independent experiments. The positions of molecular mass markers (in kDa) are indicated on the left. Notice the high efficacy of the RRAGB knock down.

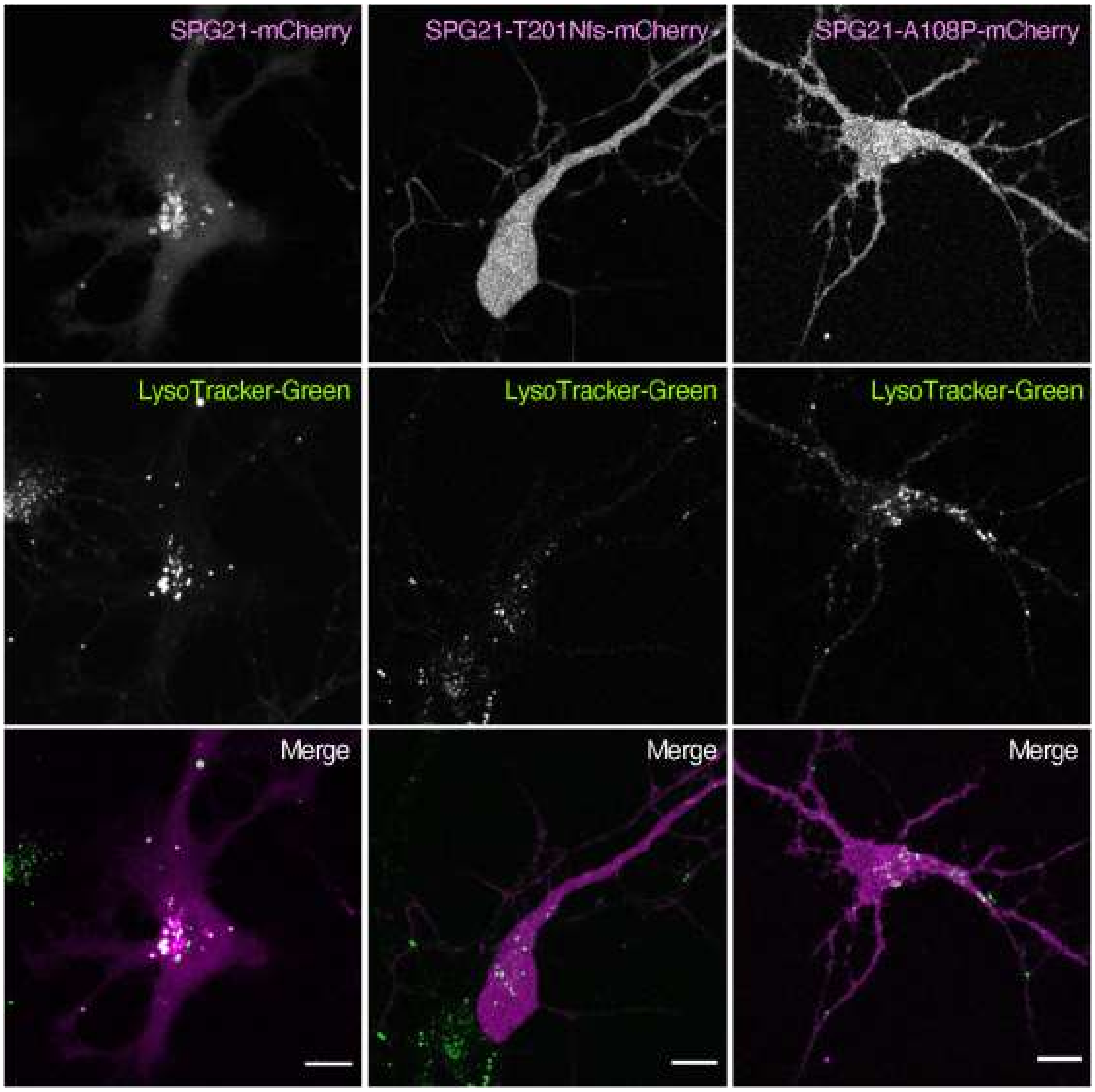

SUPPLEMENTAL FIGURE S3: SPG21 patient variants do not localize to endolysosomes in primary neurons. DIV4 rat primary hippocampal neurons in primary culture were transfected with plasmids encoding SPG21-mCherry constructs (WT, A108P, or T201Nfs) (magenta), labeled with LysoTracker Green, and imaged live by confocal fluorescence microscopy at DIV5. Scale bar: 10 μm.

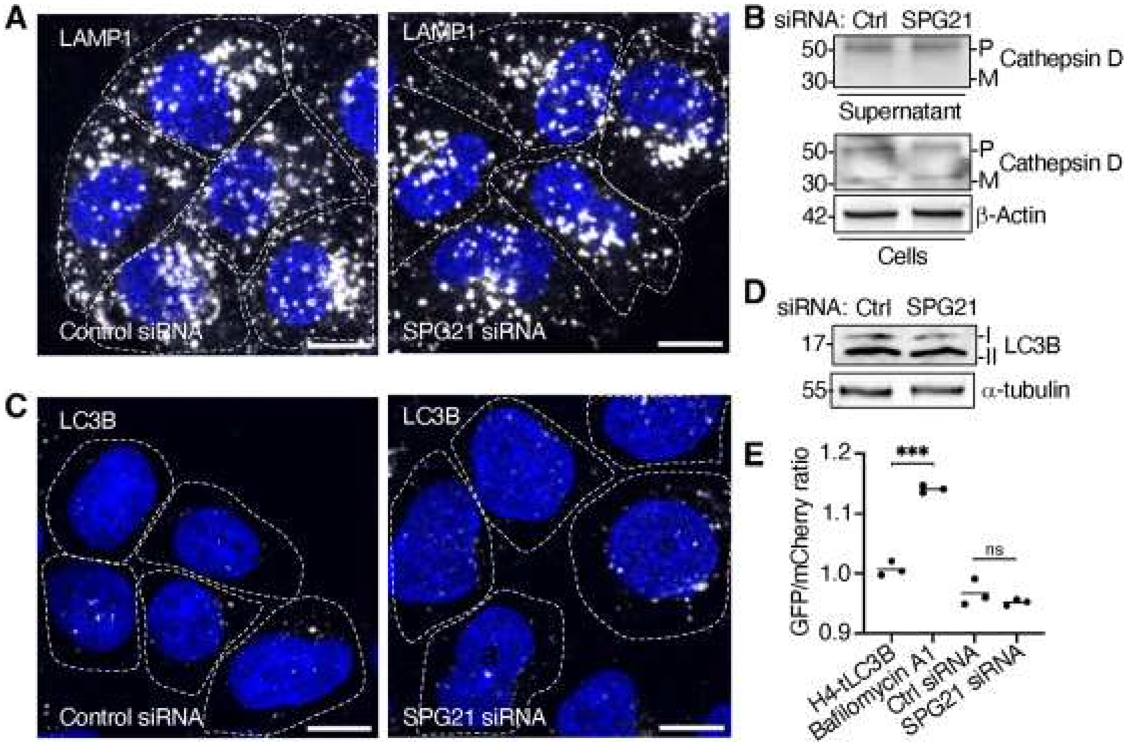

SUPPLEMENTAL FIGURE S4: Endolysosomal characteristics in SPG21-depleted cells. (A) Immunofluorescence microscopy of endogenous LAMP1 in HeLa cells treated with control or SPG21 siRNA. Nuclei were stained with DAPI (blue). Scale bars: 10 μm. (B) Immunoblot of cathepsin D in the medium and lysates of HeLa cells treated with control or SPG21 siRNA. β-actin was used as a loading control. P, precursor; M, mature. (C) Immunofluorescence microscopy of endogenous LC3B in HeLa cells treated with control or SPG21 siRNA. Nuclei were stained with DAPI (blue). Scale bars: 10 μm. (D) Immunoblot analysis of LC3B (cytosolic I and membrane-bound II forms) and α-tubulin (loading control) from lysates of HeLa cells treated with control or SPG21 siRNA. In B and D, the positions of molecular mass markers (in kDa) are indicated on the left. (E) FACS-based autophagy assay of H4 cells expressing endogenous LC3B tagged with GFP and mCherry, untreated, treated with 100 nM bafilomycin A1, control siRNA, and SPG21 siRNA. A low GFO/mCherry ratio is indicative of autophagic delivery of LC3B to lysosomes. *** $P < 0.001$, ns, not significant.
